# Data set for the proteomic inventory and quantitative analysis of chicken uterine fluid during eggshell biomineralization

**DOI:** 10.1016/j.dib.2014.09.006

**Published:** 2014-10-14

**Authors:** Pauline Marie, Valérie Labas, Aurélien Brionne, Grégoire Harichaux, Christelle Hennequet-Antier, Yves Nys, Joël Gautron

**Affiliations:** a INRA, UR83 Recherches avicoles, Fonction et Régulation des protéines de l’œuf, F-37380 Nouzilly, France; b INRA, UMR INRA85, UMR CNRS 7247, Université de Tours, IFCE, Physiologie de la Reproduction et des Comportements, Plate-forme d’Analyse Intégrative des Biomolécules, Laboratoire de Spectrométrie de Masse, F-37380 Nouzilly, France

## Abstract

Chicken eggshell is the protective barrier of the egg. It is a biomineral composed of 95% calcium carbonate on calcitic form and 3.5% organic matrix proteins. Mineralization process occurs in uterus into the uterine fluid. This acellular fluid contains ions and organic matrix proteins precursors which are interacting with the mineral phase and control crystal growth, eggshell structure and mechanical properties. We performed a proteomic approach and identified 308 uterine fluid proteins. Gene Ontology terms enrichments were determined to investigate their potential functions. Mass spectrometry analyses were also combined to label free quantitative analysis to determine the relative abundance of 96 proteins at initiation, rapid growth phase and termination of shell calcification. Sixty four showed differential abundance according to the mineralization stage. Their potential functions have been annotated. The complete proteomic, bioinformatic and functional analyses are reported in Marie et al., J. Proteomics (2015) [1].

**Table:** **Specifications table**.

Subject area	Biology
More specific subject area	Chicken uterine fluid proteome during eggshell biomineralization
Type of data	Raw and processed/analyzed mass spectrometry data obtained by nanoliquid chromatography combined to high resolution tandem mass spectrometry, .xls tables with identified/validated and quantified proteins tables
How data was acquired	LC–MS/MS using a LTQ Orbitrap Velos mass spectrometer
Data format	Raw data: raw.mzml and Processed and analyzed data using Mascot Search engine: .dat.
	Analyzed: Further assembled sequences using Clustal Omega multi-alignment algorithm and BLAST+ suite
Experimental factors	None applied
Experimental features	Uterine fluid samples were collected at three stages of mineralization process and in gel digested using trypsin. Resulting peptides were analyzed by LC–MS/MS and further treated using data mining and bioinformatic analysis
Data source location	Nouzilly, France, INRA Centre Val de Loire
Data accessibility	Data have been deposited to the ProteomeXchange Consortium Vizcaino et al., Nat. Biotechnol. 32 (2014) 223–226, via the PRIDE partner repository with the dataset identifier PXD000992 [2]

**Value of the data**

• Proteomic analysis of 308 chicken uterine fluid proteins.
• Gene ontology terms enrichments to investigate potential functions of uterine fluid proteins
• Quantitative data on protein abundances according to mineralization stage.
• Functional annotation on quantified uterine fluid proteins.

## 1 Collection of uterine fluid samples and preparation for MS analyses

Uterine fluid were collected as described previously [3] on brown-egg laying hens at 7 h, 14 h and 22 h after previous oviposition (p.o). These time intervals correspond to the initiation (I), rapid growth (G) and termination (T) phases of shell mineralization, respectively.

For global protein inventory, equal amounts of protein from uterine fluids collected at each stage of shell calcification were pooled. A total of 135 µg of proteins was fractionated on a 4–20% SDS-PAGE gel (8.3 cm×7.3 cm×1.5 mm). Proteins on gels were stained with Coomassie blue and the entire SDS-PAGE lanes were sectioned into 20 bands. For quantitative analyses, 18 individual uterine fluid samples were used. Six uterine fluids collected at the same stage were pooled in equal amount of proteins for each stage (initial (I), growth (G) and terminal (T) stage samples). The three samples I, G and T (112 µg of proteins/sample) were applied to a 4–20% SDS-PAGE gel (8.3 cm×7.3 cm×1.5 mm). A brief migration without fractionation was performed until samples were concentrated in a narrow band. The three resulting protein bands were stained with Coomassie blue and excised. Excised proteins for both approaches were in-gel digested with bovine trypsin (Roche Diagnostics GmbH, Mannheim, Germany), as previously described [4], and analyzed by nanoscale liquid chromatography-tandem mass spectrometry (nano LC–MS/MS).

## 2 Nano LC MS/MS analyses

All experiments were performed on a linear ion trap Fourier Transform Mass Spectrometer (FT-MS) LTQ Orbitrap Velos (Thermo Fisher Scientific, Bremen, Germany) coupled to an Ultimate^®^ 3000 RSLC Ultra High Pressure Liquid Chromatographer (Dionex, Amsterdam, The Netherlands) as previously described [4]. Raw data files were converted to MGF as previously described [4]. The identification of proteins was established using MASCOT search engine (v 2.3, Matrix Science, London, UK). The peptide and fragment masses obtained were matched automatically against IPI chicken (version 3.81) and against the chordata section of nr NCBI database (1601319 sequences, downloaded on 2012/03/22) and UniprotKB SwissProt (535248 sequences, downloaded on March 2012). Enzyme specificity was set to trypsin with two missed cleavages using carbamidomethylcysteine, oxidation of methionine and N-terminal protein acetylation as variable modifications. The tolerance of the ions was set to 5 ppm for parent and 0.8 Da for fragment ion matches. Mascot results obtained from the target and decoy databases searches were incorporated in Scaffold 3 software (version 3.4, Proteome Software, Portland, USA). Peptide identifications were accepted if they could be established at greater than 95.0% probability as specified by the Peptide Prophet algorithm [5]. Peptides were considered distinct if they differed in sequence. Protein identifications were accepted if they could be established at greater than 95.0% probability as specified by the Protein Prophet algorithm [6] and contained at least one sequence-unique peptide for global inventory and at least two sequence-unique peptides for quantitative analysis. A false discovery rate was calculated as <1% at the peptide or protein level. The abundance of identified proteins was estimated by calculating the emPAI using Scaffold 4 Q+ software (version 4.2, Proteome Software, Portland, USA). Additionally, to perform label-free quantitative proteomic analyses based on spectral counting method, Scaffold 3 Q+ software (version 3.4, Proteome Software, Portland, USA) was used to quantify the proteins at the three different stages of calcification. All proteins with greater than two sequence-unique peptides, identified in database with high confidence were considered for protein quantification. To eliminate quantitative ambiguity within protein groups, we ignored all the spectra matching any peptide which is shared between proteins. Thereby, quantification performed with normalized spectral counts was carried out on distinct proteins. Data have been deposited to the ProteomeXchange Consortium [2] via the PRIDE partner repository with the dataset identifier PXD000992.

## 3 Data mining and bioinformatic analysis

For proteomic inventory, a total of 256, 298 and 98 proteins were identified using nr NCBI database, IPI chicken database, and UniProtKb/Swiss-Prot database, respectively. For quantitative analysis, a total of 85 proteins were identified using the nr NCBI database, 92 proteins using the IPI chicken database, and 50 proteins with the UniProtKb/Swiss-Prot database. Data originating from both proteomic inventory and quantitative analyses were treated as follows. Trypsin and keratin from mammals were eliminated from the lists as they appeared to be contaminants or resulting from the digestion process. Protein sequences from the three databases (NCBI nr, UniprotKb SwissProt and IPI chicken) were aligned to eliminate all redundancies. Protein groups were determined using Clustal Omega multi-alignment algorithm [7]. Sequences were blasted against nr NCBI database limited to *Gallus gallus* taxon using the blastp program (BLAST+ suite) [8]. This was performed using R language (http://cran.r-project.org).

## 4 Data set analysis of avian uterine fluid proteome

A total of 308 non-redundant uterine fluid protein sequences were identified. The resulting file (Supplementary Table 1) is made of EntrezGene Ids, GI numbers, protein symbols, short descriptions, and information on their previous identification in the shell. To discern the relative abundance of these proteins among this sample, we calculated the emPAI from the proteins identified in IPI database. This calculation was made from the proteins identified in IPI database which allows us to identify the largest number of proteins. Nevertheless, a total of 35 proteins were not reported in IPI and consequently their emPAI were not determined. Furthermore, we also mentioned in Supplementary Table 1 the proteins common with those revealed in a parallel study of the uterine fluid [9]. This list of uterine fluid proteins was analysed to suppress redundant proteins and compared to our list.

In order to determine the potential functions of the 308 uterine fluid proteins, we have determined in this list, Gene Ontology terms (GO) which are widely used for the overall interpretation of the functions of proteins in proteomic or transcriptomic studies (www.geneontology.org). A total of 4277 GO terms were extracted from the 308 non-redundant uterine fluid proteins. CateGOrizer, previously known as “GO Terms Classifications Counter”, was used as a tool (http://www.animalgenome.org/tools/catego/), to analyse GO term data sets in terms of GO classes [10]. The 4277 GO terms were grouped in 98 different parent term categories using GoSlim2 method. Additionally, GO terms enrichments were determined using Gene set enrichment tools from Genomatix suite (www.genomatix.de). When considering GO terms associated to molecular functions (MF) and biological process (BP), a total of 100 GO terms were found to be significantly enriched (p-values ranges from 9.78.10^−3^ to 6.04.10^−7^), and grouped in 24 various categories (Supplementary Table 2), which represent the biological and molecular functions over-represented in the uterine fluid. The most prominent enriched group was composed of 26 proteins involved in catabolic process involving the breakdown of hexoses and the liberation of energy. Furthermore, some of these proteins were common to the enriched term associated to the chemical reactions and pathways involving small molecules. Other groups of importance were composed of uterine fluid proteins involved in protein metabolism. They corresponded to 13 proteins related to protein assembly, 7 proteins involved in folding and 19 related to protein structure and integrity. Five proteins belonging to the later were also reported in the “cellular component assembly and organization” group composed of 6 proteins. Also notable were enrichments of 6 proteins acting as protease inhibitors and 5 related to regulation of protein localization and 13 with enzyme regulator activities. Five enriched groups were associated to proteins with binding properties (cell surface-, oxygen-, lipid-, cytoskeletal- and G-protein-binding proteins). GO terms were also enriched for 13 proteins with oxido-reductase activity. Amongst them, three exhibit antioxidant activities. Oxygen transporter activity (3 proteins), transferase activity (3 proteins), response to inorganic substance (4 proteins), nutrient reservoir activity (3 proteins) and coagulation and anticoagulant activity (4 proteins) were also reported in this study. Finally, the uterine fluid proteome also exhibited an enrichment of 8 antimicrobial proteins (response to biotic stimulus).

## 5 Quantitative dataset at the three main stages of shell calcification

In a second approach, GeLC–MS/MS analyses combined to label free quantitative analysis based on spectral counting quantitative method were used to compare the respective abundance of distinct proteins at the initiation (I), rapid growth (G) and termination (T) phases of shell mineralization. A total of 96 non redundant proteins were identified. Supplementary Table 3 describes these proteins and is made of EntrezGene Ids, GI numbers, protein symbols, short descriptions, *p*-values, information on their previous identification in the shell, the calculated emPAI and their hierarchical ranking (reported from Supplementary Table 1, according to the 308 proteins constituting the uterine fluid proteome and identified in IPI database). Finally, the last three columns represent quantitative data, at the three stages of shell calcification.

Moreover, one way ANOVA was performed for protein abundance in each database, in order to reveal proteins which were significantly different between the three stages of shell mineralization. Statistically significant differences were considered for *p*-value <0.05. Amongst the 96 proteins, 64 showed differential abundance according to the three mineralization stages. Potential functions of the 64 proteins were examined according to the literature descriptions, data annotations and functional domain databases, with particular emphasis on three potential roles in the uterus during shell formation (involvement in mineralization, regulation of mineralization process and antimicrobial properties) [1]. Supplementary Table 4 reports 30 proteins that could not be ascribed to any of these three functional groups, but rather are classified as proteins with other roles.

## Conflict of interest

Authors claim no conflict of interest.

## References

[1] Marie P., Labas V., Brionne A., Harichaux G., Hennequet-Antier C, Nys Y. (2015). Quantitative proteomics and bioinformatic analysis provide new insight into protein function during avian biomineralization. J. Proteomics.

[2] Vizcaino J.A., Deutsch E.W., Wang R, Csordas A., Reisinger F., Rios D. (2014). ProteomeXchange provides globally coordinated proteomics data submission and dissemination. Nat. Biotechnol..

[3] Gautron J., Hincke M.T., Nys Y. (1997). Precursor matrix proteins in the uterine fluid change with stages of eggshell formation in hens. Connect. Tissue Res.

[4] Labas V., Grasseau I., Cahier K, Gargaros A., Harichaux G., Teixeira-Gomes A.P. (2014). Qualitative and quantitative peptidomic and proteomic approaches to phenotyping chicken semen. J. Proteomics.

[5] Keller A., Nesvizhskii A.I., Kolker E., Aebersold R. (2002). Empirical statistical model to estimate the accuracy of peptide identifications made by MS/MS and database search. Anal. Chem..

[6] Nesvizhskii A.I., Keller A., Kolker E., Aebersold R. (2003). A statistical model for identifying proteins by tandem mass spectrometry. Anal. Chem..

[7] Sievers F., Wilm A., Dineen D, Gibson T.J., Karplus K., Li W.Z. (2011). Fast, scalable generation of high-quality protein multiple sequence alignments using Clustal Omega. Mol. Syst. Biol..

[8] Camacho C., Coulouris G., Avagyan V., Ma N, Papadopoulos J., Bealer K. (2009). BLAST plus: architecture and applications. BMC Bioinf..

[9] Sun C., Xu G., Yang N. (2013). Differential label-free quantitative proteomic analysis of avian eggshell matrix and uterine fluid proteins associated with eggshell mechanical property. Proteomics.

[10] Hu Z.L., Bao J, Reecy J.M. (2008). CateGOrized: a web-based program to batch analyse gene ontology classification categories. Online J. Bioinfor..

