# Supplementary material for: Data set for the proteomic inventory and quantitative analysis of chicken uterine fluid during eggshell biomineralization
Source: Data Brief. 2014 Oct 14;1:65–9. doi: 10.1016/j.dib.2014.09.006 (PMC4459565; doi:10.1016/j.dib.2014.09.006)
Supplement: Supplementary file 1 — Supplementary data [file mmc1.zip › Table 2.docx]

**Table 2:** GO terms significantly enriched in the 308 identified uterine fluid proteins.

| **Descriptions** | **Number of proteins** | **Symbols** |
| --- | --- | --- |
| **Catabolic and carbohydrate metabolic process** | 26 | RBP4, GFPT1, PRDX6, DNASE1, ALDOC, TPI1, LDHA, LYZ, GAPDH, ENO1, PFKP, MDH1, GNAQ, PGAM1, LDHB, PGD, PRDX1, CDC42, CLU, MYH9, PKM2, UBE2V2, PGK1, GNAI2, ST3GAL1, APOA1 |
| **Small molecule metabolic process** | 25 | RBP4, CMPK1, ALDOC, TPI1, LDHA, GSTA, ASS1, GAPDH, ENO1, PFKP, MDH1, GNAQ, PGAM1, LDHB, PGD, GST, TXN, CDC42, MYH9, APOA1, CA2, PKM2, PGK1, NME2, GNAI2 |
| **Cellular aldehyde metabolic process** | 2 | RBP4, TPI1 |
| **Shell calcification** | 2 | OC-17, OC-116 |
| **Protein assembly** | 13 | RBP4, FGA, ACTR3, TUBB2C, HIST1H2B7, H3F3C, HIST2H2AC, FGB, CAPZA1, CDC42, CLU, GSN, CAPZB |
| **Protein folding** | 7 | QSOX1, CCT2, HSP90AA1, PPIB, CLU, DNAJB6, CCT8 |
| **Protein structure and integrity** | 19 | RBP4, FGA, ANXA2, ALB, ACTR3, QSOX1, GAPDH, OVOT, GNAQ, FGB, YWHAB, TXN, FN1, CAPZA1, CDC42, CLU, GSN, MYH9, CAPZB |
| **Regulation of protein localization** | 5 | RBP4, GNAQ, YWHAB, TXN, CDC42 |
| **Protease inhibitors** | 4 | OVAY, OVAL, SERPINI1, CLU |
| **Cellular component assembly and organization** | 6 | ACTR3, CAPZA1, CDC42, GSN, CAPZB, VCL |
| **Response to biotic stimulus** | 8 | RBP4, ALB, OC-17, H2B-VII, AVBD10, LYZ, OVOT, CLU |
| **Response to inorganic substance** | 4 | FGA, SOD1, FGB, PRDX1 |
| **Coagulation and anticoagulant activity** | 4 | FGA, ANXA2, ANXA5, FGB |
| **Cell surface binding** | 5 | RBP4, FGA, ANXA5, OC-17, FGB |
| **Lipid binding** | 11 | RBP4, ANXA2, ALB, ANXA11, ANXA1, APOA4, ANXA5, TENP, CRABP1, APOD, APOA1 |
| **Oxygen binding** | 4 | ALB, HBM, HBAA, HBG2 |
| **Cytoskeletal protein binding** | 11 | ANXA2, DNASE1, ACTR2, ACTR3, VCL, WDR1, CAPZA1, GSN, MYH9, DSTN, CAPZB |
| **G-protein beta/gamma-subunit complex binding** | 2 | GNAQ, GNAI2 |
| **Nutrient reservoir activity** | 3 | TPRXL, VTG2, APOVLDLII |
| **Enzyme regulator activity** | 13 | ANXA2, OVM, OVST, ANXA1, OVAY, CST3, GNAQ, OVAL, HSP90AA1, APOVLDLII, SERPINI1, OIH, OVAX |
| **Antioxidant activity** | 3 | PRDX6, SOD1, PRDX1 |
| **Oxidoreductase activity** | 13 | PRDX6, AKR1A1, QSOX1, LDHA, PLOD1, GAPDH, SOD1, MDH1, LDHB, PGD, AKR1B10, TXN, PRDX1 |
| **Oxygen transporter activity** | 3 | HBM, HBAA, HBG2 |
| **Transferase activity** | 3 | GSTA, GSTA3, GST |
