# Supplementary material for: Data set for the proteomic inventory and quantitative analysis of chicken uterine fluid during eggshell biomineralization
Source: Data Brief. 2014 Oct 14;1:65–9. doi: 10.1016/j.dib.2014.09.006 (PMC4459565; doi:10.1016/j.dib.2014.09.006)
Supplement: Supplementary file 1 — Supplementary data [file mmc1.zip › Table 4.docx]

**Table 4:** Uterine fluid proteins with no assigned functional role

| **Symbol** | **Accesion**  **IDs*** | **emPAI**** | **Description** |
| --- | --- | --- | --- |
| PSCA | 420302 | 293.6 | Prostate stem cell antigen, regulates cell proliferation |
| HBG2 | 396485 | 127.1 | Hemoglobin, gamma G, subunit of hemoglobin, involved in oxygen transport thought organism |
| HBAD | 416651 | 107.83 | Alpha-D-globin, subunit of hemoglobin, involved in oxygen transport thought organism |
| HBAA | 416652 | 94.148 | Hemoglobin, alpha 1, subunit of hemoglobin, involved in oxygen transport thought organism |
| OVAY | 420897 | 45.583 | Ovalbumin-related protein Y |
| LOC100857973 | 100857973 | 20.684 | Ganglioside GM2 activator-like |
| APOA1 | 396536 | 6.7792 | Apolipoprotein A-I, participates in the reverse transport of cholesterol from tissues to the liver |
| FOLR1 | 395638 | 5.7435 | Folate receptor 1 (adult) |
| TUBB2C | 417255 | 4.2662 | Tubulin, beta 2C, part of cytoskeleton, involved in cellular transport |
| TTR | 396277 | 3.4943 | Transthyretin, binds and transports thyroid hormones |
| PIT 54 | 395364 | 3.4845 | PIT 54 protein, probaly replaces haptoglobin in birds, transports free hemoglobin |
| LDHB | 373997 | 3.0206 | Lactate dehydrogenase B, transformes lactate into pyruvate |
| TUBA | gi\|135393 | 2.7245 | Tubulin alpha-1 chain, part of cytoskeleton, involved in cellular transport |
| GPX3 | 427638 | 2.5676 | Glutathione peroxidase 3 (plasma) catalyzes the reduction of peroxide groups by glutathione |
| SEMA3G | 415945 | 2.4926 | Sema domain, immunoglobulin domain (Ig), short basic domain, secreted, (semaphorin) 3G, inhibits axonal growth |
| ACTG1 | 415296 | 2.1116 | Actin, gamma 1, part of the cytoskeleton |
| TPI1 | 396435 | 1.559 | Triosephosphate isomerase 1, transformes D-glyceraldehyde 3-phosphate into glycerone phosphate |
| EZR | 395701 | 1.398 | Ezrin, probably involved in connections of major cytoskeletal structures to the plasma membrane |
| FAM3D | 416069 | 1.1103 | Family with sequence similarity 3, member D |
| NTM | 395450 | 0.9575 | Neurotrimin, contains immunoglobulin-like domains |
| APOH | 417431 | 0.7649 | Apolipoprotein H (beta-2-glycoprotein I) |
| VNN1 | 421702 | 0.5037 | Vanin 1, involved in the recycling of vitamin B5 |
| C4BPA | 395384 | 0.4786 | Complement component 4 binding protein, alpha, involved in the antibacterial complement cascade |
| QSOX1 | 373914 | 0.4147 | Quiescin Q6 sulfhydryl oxidase 1, catalyzes the oxidation of sulfhydryl groups in peptide and protein thiols to disulfides |
| SEMA3C | 374090 | 0.4015 | Sema domain, immunoglobulin domain (Ig), short basic domain, secreted, (semaphorin) 3C, inhibes neural growth |
| LRRC19 | 424561 | 0.3452 | Leucine rich repeat containing 19 |
| MSLN | 416534 | 0.2904 | Mesothelin |
| ABI3BP | 769237 | 0.0319 | ABI family, member 3 (NESH) binding protein, containing immunoglobulin-like fold |
| LOC423008 | 423008 | n.d. | Protein-L-isoaspartate (D-aspartate) O-methyltransferase-like |
| UBB | 396190 | n.d. | Ubiquitin B, involved in protein degradation |

* Accession ids are Entrez Gene IDs when available or protein sequence GI

** n.d. Not determined

Proteins only identified in uterine fluid
